# Immunohistochemistry Heterogeneity in Reported Breast Cancer Demographics From India: Triple-Negative Breast Cancer Rates Could Be Lower Than Suggested in Pooled Meta-Analysis

**DOI:** 10.1200/JGO.2016.006635

**Published:** 2016-09-07

**Authors:** Sanjoy Chatterjee, Indu Arun, Sanjit Agrawal, Moses Arunsingh, Indranil Mallick, Rosina Ahmed

**Affiliations:** Tata Medical Center, Kolkata, India

## To the Editor:

The meta-analysis of breast cancer demographics in Indian patients by Sandhu et al^1^ highlights significant variability in estrogen receptor (ER)/progesterone receptor (PR)/human epidermal growth factor receptor 2 (HER2) status and presenting age reported by various authors and then pools the data to ascertain the rate of triple-negative breast cancer (TNBC) to be around 31%. We feel that such a report may overlook a few important issues, which we would like to outline. The authors rightly highlight that local, environmental, and physical factors may contribute to the heterogeneity but have not explored some of them. Of the studies included, only four centers reported an average of over 20 patients per month. Small series, such as that by Akhtar et al,^2^ are likely to be unrepresentative of the regional population and could present a selection bias. However, the referral patterns for high-volume tertiary care centers may also contribute significantly to the selection bias, which is reflected in the younger age in some of these series. In addition, some studies, such as those by Nandi et al^3^ and Ambroise^4^ et al, reported immunohistochemistry (IHC) only on patients receiving curative therapy, in contrast to others, who reported on all patients who presented to the hospital. This could also contribute to the heterogeneity reported.

Some technical issues also need to be highlighted. The majority of these studies have used manual methods to determine IHC status, whereas automated methods using adequately fixed and processed tissue standardizes the technique with fewer testing variations compared with manual methods.^5^ In addition, many of the studies did not report the antibodies used or whether they followed optimal preanalytic requirements, such as cold ischemia time and adequate fixation. In most of the studies included in the analysis, IHC was performed on lumpectomies or mastectomies rather than on core biopsies; this itself may lead to a 9% false-negative ER result.^6^ Core biopsies are better specimens because of less cold ischemia time and quick formalin infiltration, resulting in uniform and consistent fixation.^7^ In addition, with the advent of robust rabbit monoclonal antibodies with improved sensitivity and specificity, such as SP1 for ER and 1E2 for PR, low levels of ER and PR are being detected, possibly reducing the number of triple-negative patients.^8^

We recently published the IHC status of unselected patients receiving curative therapy in a tertiary care center in eastern India between June 2011 and December 2013.^9^ Our overall rates of TNBC were 12.5%, with 15.5% for those with locally advanced tumors. Following the meta-analysis by Sandhu et al,^1^ we looked at our more recent data for 2014 and 2015, which showed persistent TNBC rates of 11.9% and 11.3%, respectively, with a further 5.1% and 4.4% for ER-negative/PR-negative HER2 2+ disease, where fluorescent in situ hybridization evaluation of HER2 positivity was not available. For all patients in our series, IHC was tested on mostly core biopsies using automated, approved, and peer-reviewed methods, with appropriate internal and formal external quality assurance.

The heterogeneity in the reported prevalence of TNBC and, in general, the prevalence of various luminal tumor types are likely to be multifactorial as mentioned previously. A pooled meta-analysis with the Indian-patient tag may be simplistic and may not be the actual representation, which a prospective population-based study of breast cancer with appropriate quality assurance will provide.
